# Sub-acute and acute toxicity of *Ferula asafoetida* and *Silybum marianum* formulation and effect of the formulation on delaying gastric emptying

**DOI:** 10.1186/s12906-019-2576-7

**Published:** 2019-07-05

**Authors:** Ramanaiah Illuri, Sudeep Heggar Venkataramana, David Daguet, Shyamprasad Kodimule

**Affiliations:** 1 Preclinical Research Laboratory, R&D Center for Excellence, Vidya Herbs Pvt. Ltd, AnekalTaluk, Jigani Industrial Area, #14A, KIADB, Jigani I phase, Bangalore, Karnataka 560 105 India; 2 Vidya Europe SAS, 7 avenue de Norvège, 91140 Villebon sur Yvette, France

**Keywords:** Herbal formulation, Dyspepsia, Safety, Rats

## Abstract

**Background:**

Delayed gastric emptying play an important role in the pathology of functional dyspepsia. Owing to their functional attributes in alleviating the gastrointestinal disorders, single or polyherbal formulations have gained attention to treat the symptoms of functional dyspepsia. We have investigated the safety and efficacy of a novel formulation of *Ferula asafoetida* oleo resin and standardized *Silybum marianum* extract (Asdamarin).

**Methods:**

The effect of asdamarin on delayed gastric emptying was investigated in Sprague Dawley rats using phenol red method. The acute and sub-acute oral toxicity was evaluated in wistar rats following OECD guidelines 425 and 407 respectively. The data were analyzed by one-way ANOVA using GraphPad Prism 5.0 software.

**Results:**

Oral administration of Asdamarin dose-dependently improved the delay in gastric emptying as evident from the significant increase in the gastrointestinal transit time (*p* < 0.001). The LD50 of asdamarin was estimated to be more than 2000 mg/kg. Further, in the 28-day sub-acute toxicity study, the administration of 250, 500 and 1000 mg/kg of Asdamarin did not significantly altered the feed and water consuption, body weight change, biochemical and haematological parameters compared to control animals. Macroscopic and histopathological examination of vital organs revealed no toxic signs.

**Conclusion:**

The preliminary data from the present study provides the first evidence on the possible effectiveness of novel formulation of *F*. *Asafoatida* and *S. marianum* extracts in alleviating the associated symptoms of functional dyspepsia. The toxicity data indicated that Asdamarin can be considered safe up to 1000 mg/kg dose.

## Background

Dyspepsia is an umbrella term used to characterize abdominal pain centered in the epigastrium, sometimes combined with other gastrointestinal complaints. Functional dyspepsia is a common gastrointestinal disorder associated with decrement in the quality of life [1]. FD is characterized majorly by the disturbances in the gastric emptying and motility [2]. There is accumulating evidence that distinct subgroups of uninvestigated dyspepsia exist in the general population, suggesting the requirement for separate evaluation and treatment strategies [3, 4]. Treatments for FD include acid suppressing medicines (proton pump inhibitors), Selective Serotonin re-uptake inhibitors (SSRIs) and drugs affecting gastric motility such as domperidone and mosapride [5]. Medicinal plant preparations have gained increasing attention in the treatment of FD due to their potential health benefits and safety [5–7]. Most of the herbal remedies for treating FD symptoms worldwide are combinations of several medicinal plants [8].

*Ferula asafoetida* belonging to the family *Umbelliferae* is a perennial plant valued for its oleo-gum-resin (exudates obtained from the rhizome) used in traditional medicine in different parts of the world for many treating several ailments which include treatment of conditions such as asthma, bronchitis, stomach ache, ulcer, intestinal parasites and epilepsy [9–11]. Asafoetida is used mainly for the stomach related ailments and digestion in Ayurveda. Higwastaka, a popular polyherbal formulation of Asafoetida is used as digestive. Asafoetida along with other dietary spices facilitates digestion by promoting the activities of digestive enzymes in pancreas and small intestine, stimulating the bile acid production [12]. Asafoetida in combinational herbal preparations has been studied extensively for stomach related ailments. Gopi et al. showed that asafoetida encapsulated in turmeric nanofibers demonstrated positive effects by attenuating the disease activity in a rat model of ulcerative colitis [13]. A combination of asafoetida and fenugreek fibres exhibited significant stomach protection in ethanol-induced ulcer model rats [14]. Further, in vitro experiment on guinea pig and rat isolated ileums demonstrated the antispasmodic activity of asafoetida [15, 16].

*Silybum marianum* (Fam. Asteriaceae) commonly known as milk thistle is a medicinal plant valued for its potential health benefits long since. Experimental and clinical evidence suggest that the plant is used extensively for treating liver disorders due to its potential antioxidant and hepatoprotective effects [17]. It has been also studied for hypoglycemic and antidiabetic activities [18]. The pharmacological benefits of milk thistle have been largely studied in various experimental models. Milk thistle exerted anti-inflammatory effects in NASH model rats [19]. In another study, rats fed with high fat diet exhibited significant amelioration of non-alcoholic fatty liver disease (NAFLD) following a 6-week treatment with silybin, the active principle of milk thistle [20].

Animal models have been extensively used to study various aspects of FD, and to identify therapeutic interventions [21]. Delayed gastric emptying is one of the major pathophysiological disturbance of FD which can be studied using animal models such as rodents [22]. Here we have evaluated the effect of a combination of asafoetida and *S. marianum* extracts (Asdamarin) on gastrointestinal motility using in vivo model rats. Asdamarin is a proprietary blend of *F. asafoetida* (oleo-gum-resin) CO_2_ extract and *S. marianum* (milk thistle) extract in the ratio of 1:3. The present study was designed to evaluate the efficacy of Asdamarin in improving the delayed gastric emptying, and to investigate it’s in vivo toxicity.

## Methods

### Asdamarin

The investigational herbal formulation, Asdamarin was supplied by the Department of Quality Control, Vidya Herbs (P) Ltd.

### High performance liquid chromatography (HPLC) analysis

The HPLC analysis was performed on a C18 column (4.6 × 150 mm, Phenomenex Kinetex) at a UV detection of 288 nm (HPLC-LC 2010HT). The mobile phase of methanol/0.5% phosphoric acid/water was flowed at 1.0 mL/min through the column.

### Chemicals and animals

Phenol red powder were purchased from Sigma (St. Louis, MO). Wistar rats (6–8 weeks) and male Sprague Dawley (SD) rats (190–200 g) were procured from authorized suppliers of laboratory animals – Biogen, Bangalore, India (Reg No. 971/PO/RcBiBt/S/2006/CPCSEA). The animals were placed in polypropylene cages and housed in a room under controlled atmosphere (temperature, 22 ± 3 °C, humidity, 30–70%; 12 h light/dark cycle). During a 1-week acclimatization period, all rats consumed a commercial diet and tap water ad libitum. The animal studies were performed after due clearance from the Institutional Animal Ethics Committee (VHPL/PCL/IAEC/05/18) independently formed by CPCSEA (Committee for the purpose of control and supervision of experiments on animals, a statutory committee established under the Prevention of Cruelty to Animals Act, 1960 in India).

### Determination of gastric emptying by phenol red method

Twenty-four male SD rats were divided into four groups of six animals each. Group I was control group administered with physiological saline; Group II animals were given physiological saline for seven days and on the 8th day reference drug Domiperidone was administered. Group III and IV were administered orally with two test doses of Asdamarin (50 and 100 mg/kg) for seven days. On day 8, 2 h later to the respective treatments gastric emptying was measured using phenol red method as described previously [1]. 18-h fasted rats were administered intragastrically with 1.5% carboxymethyl cellulose sodium salt containing 0.05% phenol red (0.5 mL/mouse). Rats were sacrificed after 20 min by ketamine/xylazine (80 mgkg^− 1^/10 mg/kg^− 1^) overdose. Stomach was harvested, and gastric content collected. The gastric content was treated with 10 mL of 0.1 M NaHCO_3_ and centrifuged at 3000 rpm for 15 min. The amount of phenol red in the supernatant was determined based on the absorbance at 570 nm measured using a microplate reader (Multiskan EX, Thermo Scientific). The amount of phenol red from an animal sacrificed immediately after the above-mentioned administration procedure was used as the standard sample. Gastric emptying was calculated using the formula: (1-amount of phenol red in the test sample/amount of phenol red in the standard sample) × 100. Percentage of gastrointestinal transit time (GIT) was determined using the formula: (Total length of small intestine of rat/Length of phenol red movement in the intestine) × 100.

### Acute oral toxicity of Asdamarin

Asdamarin was evaluated for acute oral toxicity at the dose level of 2000 mgkg^− 1^ in accordance with OECD (Organization for Economic Cooperation and Development) guideline 425 [23]. Five female wistar rats were housed in a cage prior to dosing. A limit test was performed where the first animal was administered orally with the upper limit dose of 2000 mgkg^− 1^ b.w. and observed for 24 h. Depending upon the survival, other four animals were given the limit dose and observed for mortality. The animals were observed individually after dosing once during the first 30 min, periodically during the first 24 h, with special attention given during the first 4 h, and daily thereafter for a total of 14 days.

### Sub-acute oral toxicity

Fifty (25 male and 25 female) healthy wistar rats aged 6–8 weeks were used for evaluating the sub-acute oral toxicity of Asdamarin. The study was performed in compliance with OECD guideline No. 407 [24]. The animals were divided into five groups of 10 animals each (5 males and 5 females). Group I animals received 0.9% normal saline (control), group II, III and IV received 250, 500 and 1000 mgkg^− 1^asdamarin on daily basis for 28 days. In order to monitor reverse sign of any toxicity a satellite group was included as group V. This group was administered with 1000 mgkg^− 1^ Asdamarin daily for 28 days, and there was no further treatment for 14 days before the end of study. Visual observations for mortality, behavioural pattern and clinical signs of illness were made daily during the study period. Body weight of animals in each group was assessed on weekly intervals and, feed and water consumption were assessed daily for the entire period. At the end of study, overnight fasted rats were anesthetized by ketamine/xylazine (80 mgkg^− 1^/10 mg/kg^− 1^) overdose. Blood samples of the animals were collected via cardiac puncture in tubes containing ethylenediaminetetraacetic acid (EDTA) and tested for haematological and biochemical parameters. The organs were excised, and relative weights of vital organs were determined using the formula, 100 × (organ weight/body weight).

Major organs such as liver, kidneys, brain, spleen and heart were preserved in 10% buffered formalin for histological examination. The tissue samples were fixed in 4% formalin, dehydrated with a graded alcohol series, embedded in paraffin, and then cut into 5 μm thickness. The sections were stained with hematoxylin and eosin (H&E, Sigma Aldrich, St. Louis, MO, USA). The images were captured using a microscope (Leica, Germany).

### Statistical analysis

The data were analyzed using GraphPad Prism version 5.0. The values were recorded as mean ± s.e.m. and analyzed statistically using one-way ANOVA followed by Tukey test. *p* < 0.05 was considered statistically significant.

## Results

### HPLC analysis of Asdamarin

**Fig. 1:**
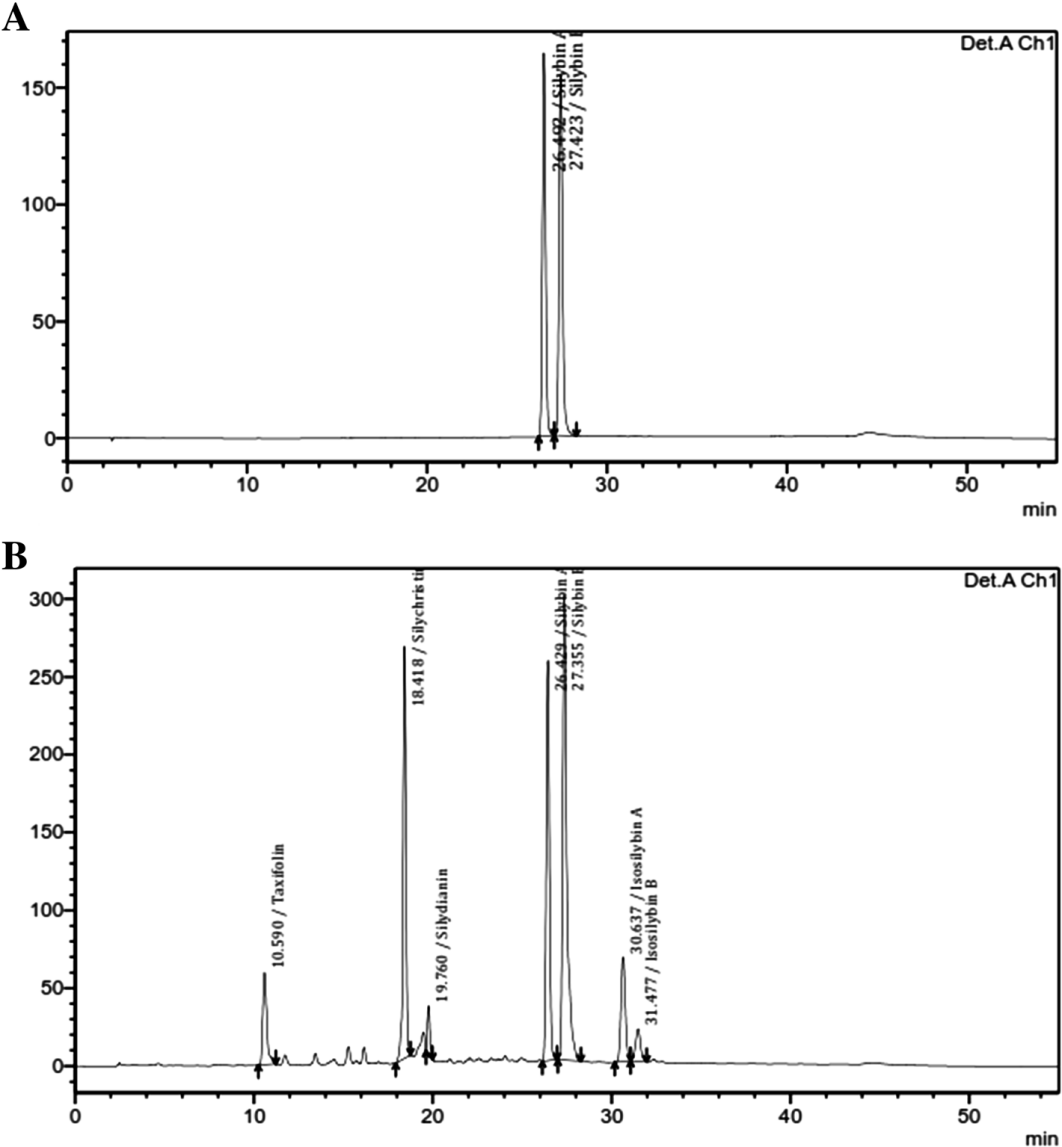
HPLC chromatogram of (**a**) Silibinin (98%) and (**b**) Asdamarin

Asdamarin was characterized for the presence of silymarin (not less than 25%). It contains combination of flavonoids such as Silybin A, Silybin B, Taxifolin, Silychristin, Silydianin, Isosilybin A and Isosilybin B (Fig. 1).

### Effect of Asdamarin on gastric emptying

**Fig. 2:**
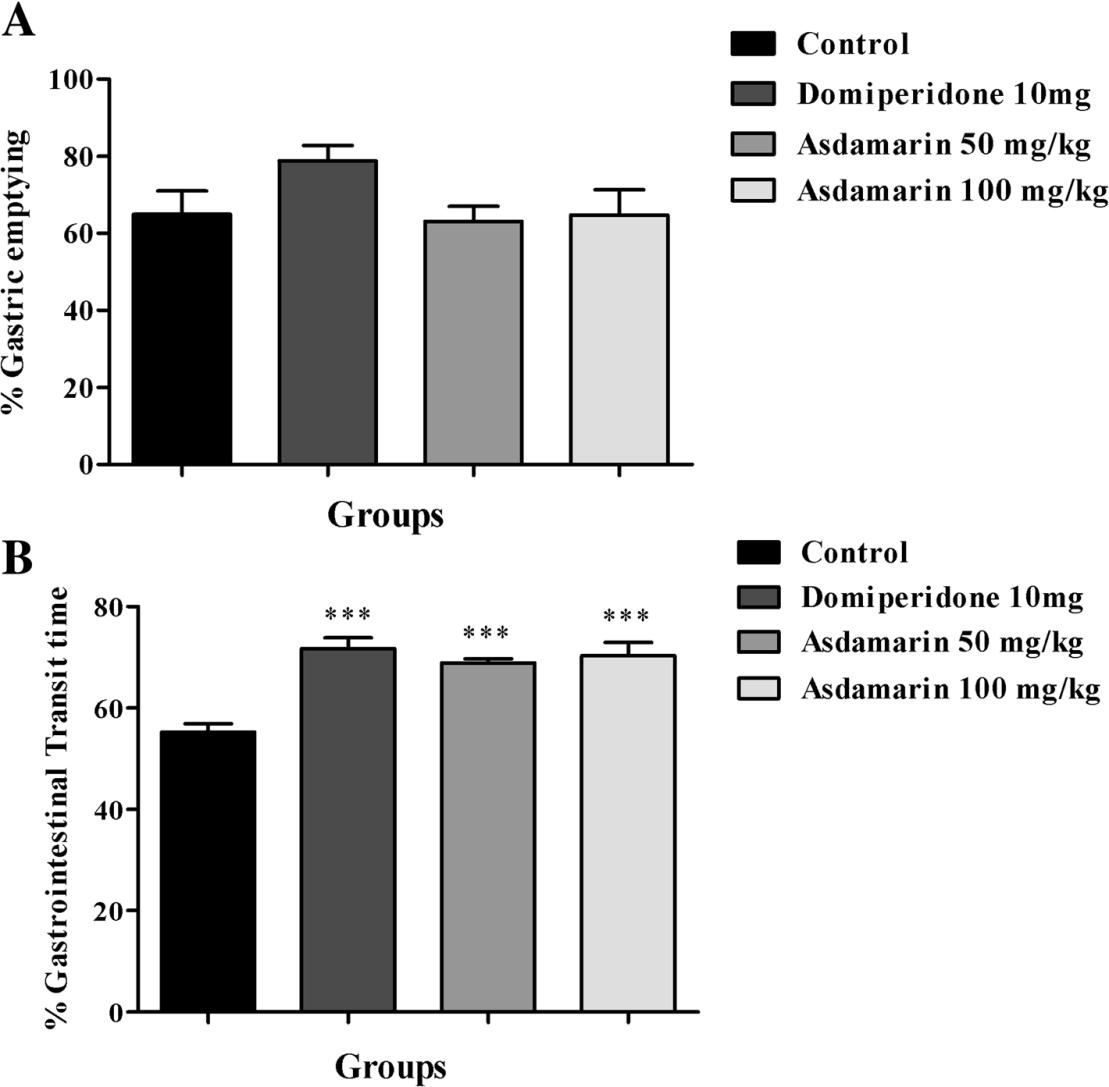
Effect of Asdamarin on (**a**) gastric emptying and (**b**) percentage gastrointestinal transit time (GIT) in SD rats. Values are expressed as mean ± SEM (*n* = 6). Data were analyzed by one way ANOVA followed by Dunnet’s t test. ***p < 0.001 compared to control group

The percentage gastric emptying was assessed after 20 min of phenol red meal administration in rats. The gastric emptying was lower in control animals (64.94%) while the animals in Domperidone treated group showed an increase in percentage of gastric emptying (78.86%). Rats treated with 50 and 100 mg/kg doses of Asdamarin exhibited increased percentage of gastric emptying (69.8 and 74.79% respectively) as compared to control (Fig. 2a). However, the data were not statistically significant.

Further, in the present study gastrointestinal transit (GIT) was found to be increasing significantly in the extract and standard drug treated rats as compared to control. The GIT was 55.19% in control group. There was a significant increase in GIT among the animals treated with single dose of Domperidone (*p* < 0.001). Asdamarin administered rats showed significant improvement in the GIT dose dependently (*p* < 0.001). The GIT was 68.9 and 70.28% respectively for 50 and 100 mg/kg Asdamarin (Fig. 2b).

### Acute oral toxicity

In the present study, single dose administration of Asdamarin (*p.o.*) in female rats at 2000 mg/kg did not induce any changes in the behavioural, motor and neuronal functions. Asdamarin treatment had no effect on mortality, body weight change or gross observation. Hence, the lethal dose of Asdamarin might be higher than 2000 mg/kg.

### Sub-acute oral toxicity

#### Body weight, food and water consumption

**Fig. 3:**
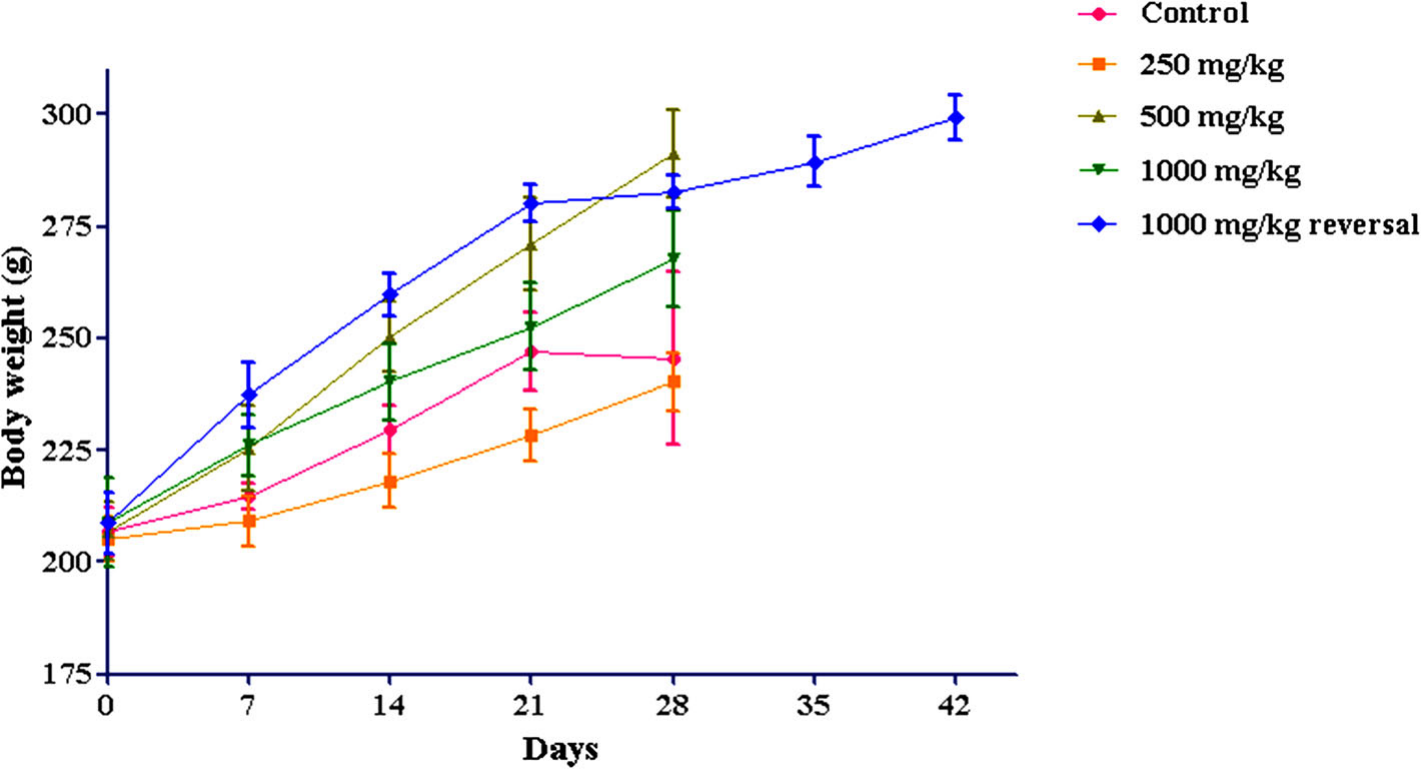
Effect of 28-day treatment with Asdamarin. on body weight of male rats. Values are expressed as mean ± s.e.m. (n = 5). *p < 0.05 were considered significant using one-way Anova

**Fig. 4:**
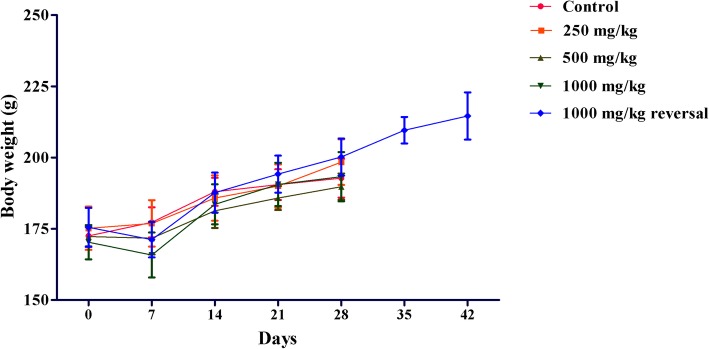
Effect of 28-day treatment with Asdamarin on body weight of female rats. Values are expressed as mean ± s.e.m. (*n* = 5). *p < 0.05 were considered significant using one-way Anova

28-day oral administration of Asdamarin did not alter the feed and water consumption in rats compared to the respective control animals (Tables 1 and 2). Further there was neither mortality nor significant changes in the body weights of rats treated with 250–1000 mg/kg Asdamarin in comparison with control group (Figs. 3 and 4). Physical observations indicated no toxic signs in the fur, skin, eyes, tremors, salivation and behavioural patterns of rats at the tested doses. No abnormal gross findings were observed in the necropsies of Asdamarin treated rats at all the test doses. Overall, no adverse events were recorded during the toxicity evaluation of Asdamarin.

**Table 1:** Effect of Asdamarin on mean feed consumption (g) in rats

Sex	Day	Control	250 mg/kg	500 mg/kg	1000 mg/kg	1000 mg/kg reversal
Male	7	17.83 ± 0.95	16.8 ± 0.42	18.31 ± 0.7	18.19 ± 0.61	21.7 ± 0.86
	14	18.8 ± 0.36	17.72 ± 0.93	22.13 ± 0.7	20.12 ± 0.58	24.36 ± 0.47
	21	20.84 ± 0.46	19.3 ± 0.24	24.64 ± 0.57	21.44 ± 0.72	23.92 ± 0.14
	28	21.54 ± 1.03	20.09 ± 0.47	24.03 ± 0.54	22.81 ± 0.69	23.3 ± 0.66
	35	–	–	–	–	19.78 ± 0.29
	42	–	–	–	–	23.95 ± 0.35
Female	7	14.62 ± 0.30	14.18 ± 0.46	13.86 ± 0.35	12.61 ± 0.90	14.00 ± 0.82
	14	15.83 ± 0.57	16.43 ± 0.48	15.15 ± 0.33	17.17 ± 0.34	16.90 ± 1.27
	21	15.77 ± 0.87	15.81 ± 0.33	15.11 ± 0.54	17.12 ± 0.30	17.63 ± 0.28
	28	16.35 ± 0.68	17.74 ± 0.68	17.22 ± 0.39	18.89 ± 0.52	19.63 ± 1.11
	35	–	–	–	–	20.69 ± 0.49
	42	–	–	–	–	18.88 ± 1.08

Values are expressed as mean ± s.e.m. (*n* = 10 for each group). Data were analyzed by one-way Anova. *p < 0.05 were considered as statistically significant compared to control

**Table 2:** Effect of Asdamarin on average water consumption (mL) in rats

Sex	Day	Control	250 mg/kg	500 mg/kg	1000 mg/kg	1000 mg/kg reversal
Male	7	28.46 ± 0.94	27.49 ± 0.99	35.71 ± 1.08	30.8 ± 1.15	36.63 ± 1.81
	14	34.46 ± 1.35	32.46 ± 3.27	39.54 ± 2.68	36.51 ± 0.96	38.57 ± 2.43
	21	35.46 ± 2.50	30.57 ± 1.10	44.74 ± 0.65	41.69 ± 1.53	39.06 ± 1.14
	28	30.69 ± 1.46	32.74 ± 1.49	30.14 ± 0.55	42.8 ± 1.72	38.66 ± 1.79
	35	–	–	–	–	37.09 ± 2.27
	42	–	–	–	–	36.94 ± 1.05
Female	7	30.51 ± 1.14	25.77 ± 1.19	26.46 ± 2.84	29.29 ± 1.31	27.63 ± 3.13
	14	29.6 ± 29.69	24.4 ± 28.03	28.8 ± 30.11	33.2 ± 35.54	28.00 ± 29.09
	21	32.00 ± 29.89	27.00 ± 24.17	27.6 ± 25.60	35.00 ± 34.17	36.2 ± 30.14
	28	32.83 ± 2.83	36.8 ± 2.43	35.94 ± 1.91	38.26 ± 2.20	37.11 ± 2.06
	35	–	–	–	–	40.31 ± 3.37
	42	–	–	–	–	27.17 ± 2.13

Values are expressed as mean ± s.e.m. (*n* = 10 for each group). Data were analyzed by one-way Anova. *p < 0.05 were considered as statistically significant compared to control

#### Relative organ weights

The results of relative organ weight measurement are shown in Table 3. The 28-day treatment with Asdamarin did not significantly alter the relative organ weights of male and female rats compared to the control group.

**Table 3:** Relative organ weights of rats treated with Asdamarin for 28 days

Sex	Organ	Control	Asdamarin (mg/kg, B.W)			
			250 mg/kg	500 mg/kg	1000 mg/kg	1000 R mg/kg
Male	Brain	0.808 ± 0.05	0.795 ± 0.03	0.659 ± 0.006*	0.714 ± 0.042	0.686 ± 0.014
	Heart	0.368 ± 0.03	0.337 ± 0.01	0.308 ± 0.006	0.326 ± 0.016	0.303 ± 0.004
	Liver	3.613 ± 0.14	3.360 ± 0.13	3.511 ± 0.126	3.815 ± 0.126	2.456 ± 0.047
	Spleen	0.486 ± 0.09	0.44 ± 0.02	0.399 ± 0.036	0.447 ± 0.04	0.392 ± 0.03
	Kidneys	0.339 ± 0.01	0.335 ± 0.02	0.308 ± 0.019	0.341 ± 0.015	0.284 ± 0.009
Female	Brain	0.930 ± 0.033	0.858 ± 0.022	0.914 ± 0.028	0.913 ± 0.019	0.874 ± 0.032
	Heart	0.403 ± 0.026	0.388 ± 0.021	0.385 ± 0.0187	0.396 ± 0.007	0.336 ± 0.013
	Liver	3.349 ± 0.331	3.199 ± 0.150	3.054 ± 0.228	3.150 ± 0.089	2.627 ± 0.084
	Spleen	0.520 ± 0.061	0.465 ± 0.076	0.448 ± 0.036	0.523 ± 0.045	0.325 ± 0.026
	Kidneys	0.503 ± 0.025	0.515 ± 0.015	0.505 ± 0.022	0.521 ± 0.008	0.455 ± 0.024

Values are expressed as mean ± s.e.m. (n = 10 for each group). *p < 0.05 were considered significant using one-way Anova. * denote significant difference compared to control

#### Haematology and clinical biochemistry analysis

The effect of 28-day treatment with Asdamarin on haematological parameters is presented in Table 4. Except for the marginal changes in some of the parameters, the haematological assessment showed no significant change in the treatment groups as compared to control. Further, Asdamarin administration exerted no significant changes in the biochemical analyses such as renal (urea and creatinine) and liver function (alanine aminotransferase, aspartate aminotransferase and alkaline phosphatase) parameters, total protein and albumin (Table 5).

**Table 4:** Effect of Asdamarin on haematological parameters in rats

	Unit	Control	Asdamarin (mg/kg, B.W)			
			250	500	1000	1000 Reversal
Male						
Haemoglobin	g/dL	16.78 ± 0.56	17.60 ± 0.27	15.40 ± 0.66	16.06 ± 0.56	16.26 ± 0.51
RBC	10^6^/μL	9.848 ± 1.25	8.55 ± 0.15	7.72 ± 0.24	7.97 ± 0.3	7.97 ± 0.27
HCT	%	43.98 ± 0.66	45.66 ± 0.53	40.40 ± 1.08	41.24 ± 1.84	41.00 ± 1.26
MCV	fL	51.98 ± 1.03	53.54 ± 1.16	52.48 ± 1.33	51.80 ± 0.85	51.52 ± 0.82
MCH	Pg	19.74 ± 0.39	20.56 ± 0.41	19.86 ± 0.3	20.12 ± 0.20	20.34 ± 0.32
MCHC	g/dL	38.10 ± 1.06	38.50 ± 0.22	38.04 ± 1.06	38.96 ± 0.43	39.60 ± 0.35
Platelets	10^3^/μL	270.6 ± 21.78	307.4 ± 16.85	313.0 ± 16.88	313.6 ± 10.16	311.4 ± 20.48
WBC	10^3^/μL	22.06 ± 0.87	19.22 ± 3.12	19.46 ± 1.29	19.62 ± 2.76	20.04 ± 2.11
Lymphocytes	%	90.92 ± 1.66	88.74 ± 0.88	93.84 ± 0.62	93.94 ± 0.76	90.20 ± 1.47
Monocytes	%	3.18 ± 0.41	3.42 ± 0.19	2.35 ± 0.16	2.27 ± 0.14	2.97 ± 0.30
Neutrophils	%	4.54 ± 1.08	6.38 ± 0.7	2.80 ± 0.41	2.82 ± 0.56	5.56 ± 1.08
Eosinophils	%	1.33 ± 0.17	1.43 ± 0.07	0.99 ± 0.07	0.95 ± 0.06	1.25 ± 0.12
Basophils	%	0.06 ± 0.02	0.06 ± 0.02	0.06 ± 0.02	0.06 ± 0.02	0.06 ± 0.02
Clotting time	Seconds	33.0 ± 14.59	32.60 ± 4.49	39.80 ± 21.67	25.00 ± 4.50	27.40 ± 5.97
Female						
Haemoglobin	g/dL	16.68 ± 0.57	15.48 ± 0.30	16.64 ± 0.466	15.50 ± 0.59	15.42 ± 0.34
RBC	10^6^/μL	7.57 ± 0.26	7.67 ± 0.21	7.13 ± 0.24	6.93 ± 0.62	7.45 ± 0.10
HCT	%	43.98 ± 1.66	39.22 ± 0.53	38.78 ± 1.03	36.84 ± 3.15	39.36 ± 0.72
MCV	fL	58.14 ± 1.22	54.28 ± 1.18	56.74 ± 0.83	60.32 ± 1.09	56.90 ± 0.33
MCH	Pg	22.00 ± 0.29	20.16 ± 0.39	20.48 ± 0.31	21.38 ± 1.59	20.64 ± 0.27
MCHC	g/dL	37.90 ± 0.36	39.42 ± 0.26	39.74 ± 0.31	40.18 ± 2.81	39.14 ± 0.35
Platelets	10^3^/μL	310.8 ± 40.08	345.2 ± 12.87	300.0 ± 28.68	338.8 ± 41.25	272.8 ± 18.13
WBC	10^3^/μL	11.02 ± 1.71	8.28 ± 1.25	8.40 ± 1.38	9.36 ± 1.53	9.36 ± 1.58
Lymphocytes	%	91.28 ± 1.66	94.30 ± 0.24	94.64 ± 0.79	93.04 ± 0.58	92.18 ± 1.82
Monocytes	%	2.954 ± 0.52	2.18 ± 0.11	2.19 ± 0.34	2.016 ± 0.24	2.70 ± 0.52
Neutrophils	%	4.50 ± 0.97	2.58 ± 0.12	3.22 ± 0.33	3.08 ± 0.28	3.96 ± 1.21
Eosinophils	%	1.25 ± 0.22	0.92 ± 0.04	0.92 ± 0.15	0.85 ± 0.10	1.13 ± 0.21
Basophils	%	0.06 ± 0.02	0.06 ± 0.02	0.06 ± 0.02	0.06 ± 0.02	0.06 ± 0.02
Clotting time	Seconds	64.2 ± 19.90	33.0 ± 5.46	26.40 ± 3.09	39.00 ± 11.79	53.80 ± 14.61

Values are expressed as mean ± s.e.m. (n = 10 for each group). *p < 0.05 were considered significant using one-way Anova

**Table 5:** Effect of Asdamarin on serum biochemical parameters in rats

	Unit	Control	Asdamarin (mg/kg)			
			250	500	1000	1000 Reversal
Male						
ALT	IU/L	209.2 ± 69.38	212.0 ± 60.64	146.8 ± 7.14	219.0 ± 46.85	66.0 ± 6.88
AST	IU/L	296.9 ± 53.02	279.6 ± 43.44	228.6 ± 10.88	238.8 ± 25.25	208.4 ± 20.78
ALP	IU/L	276.9 ± 39.06	246.1 ± 5.54	221.4 ± 29.71	207.2 ± 18.54	299.3 ± 39.60
Total Protein	g/dL	6.20 ± 0.39	12.08 ± 3.37	8.0 ± 1.36	5.90 ± 1.33	8.52 ± 0.46
Albumin	mg/dL	3.60 ± 0.19	4.58 ± 0.76	5.24 ± 1.37	6.62 ± 1.59	4.06 ± 0.25
Glucose	mg/dL	115.7 ± 6.16	135.1 ± 7.95	92.22 ± 16.19	131.3 ± 19.92	114.9 ± 10.15
Total bilirubin	mg/dL	0.24 ± 0.24	0.24 ± 0.02	0.26 ± 0.04	0.74 ± 0.31	0.26 ± 0.024
Direct bilirubin	mg/dL	0.12 ± 0.02	0.10 ± 0.03	0.160 ± 0.04	0.14 ± 0.05	0.120 ± 0.02
Urea	mg/dL	37.43 ± 5.72	35.17 ± 3.32	33.83 ± 3.83	48.17 ± 4.30	54.67 ± 6.33
Creatinine	mg/dL	0.64 ± 0.07	0.79 ± 0.31	2.18 ± 1.14	1.74 ± 0.91	0.84 ± 0.05
Cholesterol	mg/dL	53.03 ± 7.22	57.13 ± 5.34	63.78 ± 2.80	58.57 ± 21.28	78.85 ± 6.40
Triglycerides	mg/dL	52.57 ± 14.57	51.34 ± 10.32	66.43 ± 9.29	58.75 ± 20.85	70.86 ± 6.37
HDL	mg/dL	43.08 ± 4.87	47.99 ± 3.80	50.11 ± 1.60	53.53 ± 6.99	68.72 ± 5.94**
Calcium	mg/dL	9.87 ± 0.78	19.37 ± 9.54	12.60 ± 3.15	14.56 ± 4.40	10.96 ± 0.55
Phosphorous	mg/dL	17.32 ± 6.07	24.04 ± 6.85	22.38 ± 3.1	26.08 ± 11.58	21.34 ± 16.60
Female						
ALT	IU/L	74.40 ± 4.88	105.0 ± 15.44	101.4 ± 10.46	108.6 ± 16.59	52.2 ± 7.14
AST	IU/L	170.6 ± 7.69	180.4 ± 15.46	178.5 ± 17.91	189.0 ± 23.67	175.5 ± 9.74
ALP	IU/L	160.0 ± 17.89	190.5 ± 31.15	142.7 ± 6.46	184.6 ± 34.12	165.4 ± 18.7
Total Protein	g/dL	13.90 ± 1.24	14.1 ± 1.56	11.48 ± 1.00	11.84 ± 1.59	10.58 ± 1.14
Albumin	mg/dL	4.08 ± 0.08	3.86 ± 0.09	3.720 ± 0.31	3.82 ± 0.198	3.66 ± 0.19
Glucose	mg/dL	107.7 ± 9.17	117.8 ± 22.54	121.1 ± 11.32	110.1 ± 34.12	109.9 ± 6.27
Total bilirubin	mg/dL	0.28 ± 0.04	0.34 ± 0.14	0.36 ± 0.09	0.26 ± 0.07	0.32 ± 0.037
Direct bilirubin	mg/dL	0.16 ± 0.02	0.18 ± 0.08	0.24 ± 0.05	0.14 ± 0.02	0.16 ± 0.02
Urea	mg/dL	123.2 ± 13.82	107.8 ± 17.75	150.7 ± 36.99	130.7 ± 12.54	51.49 ± 3.86
Creatinine	mg/dL	0.43 ± 0.04	1.09 ± 0.66	1.83 ± 1.21	1.04 ± 0.17	0.69 ± 0.1
Cholesterol	mg/dL	69.58 ± 8.39	55.86 ± 4.96	53.24 ± 6.20	63.03 ± 8.54	61.76 ± 3.76
Triglycerides	mg/dL	65.15 ± 12.15	44.65 ± 13.84	53.12 ± 10.79	44.05 ± 23.35	101.7 ± 29.26
HDL	mg/dL	64.70 ± 5.65	60.44 ± 5.52	62.44 ± 6.70	68.13 ± 3.76	74.76 ± 3.92
Calcium	mg/dL	9.41 ± 0.19	8.44 ± 0.42	9.54 ± 0.90	7.35 ± 2.65	9.33 ± 0.13
Phosphorous	mg/dL	23.02 ± 2.88	14.90 ± 5.17	13.28 ± 5.31	22.70 ± 6.80	24.42 ± 4.2

Values are expressed as mean ± s.e.m. (*n* = 10 for each group). *p < 0.05 were considered significant using one-way Anova. **p < 0.01 Vs. control

#### Histopathology

**Fig. 5:**
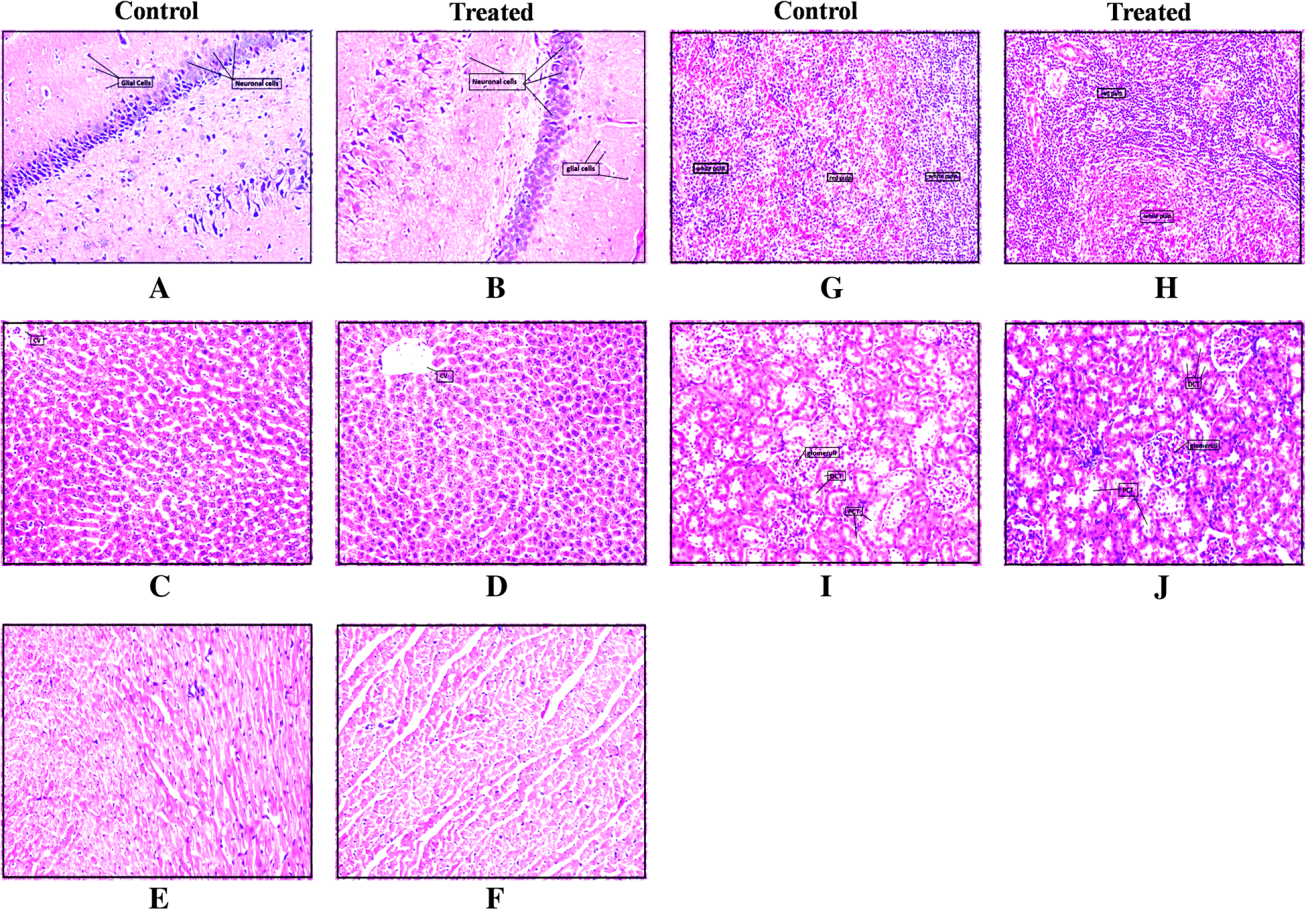
Effect of 1000 mg kg^−1^of Asdamarin on histology of vital organs of male rats. **a** and (**b**): brain; (**c**) and (**d**): liver; (**e**) and (**f**): heart; (**g**) and (**h**): spleen; (**i**) and (**j**): kidney. cv, central vein; DCT, distal convoluted tubule; PCT, proximal convoluted tubule

**Fig. 6:**
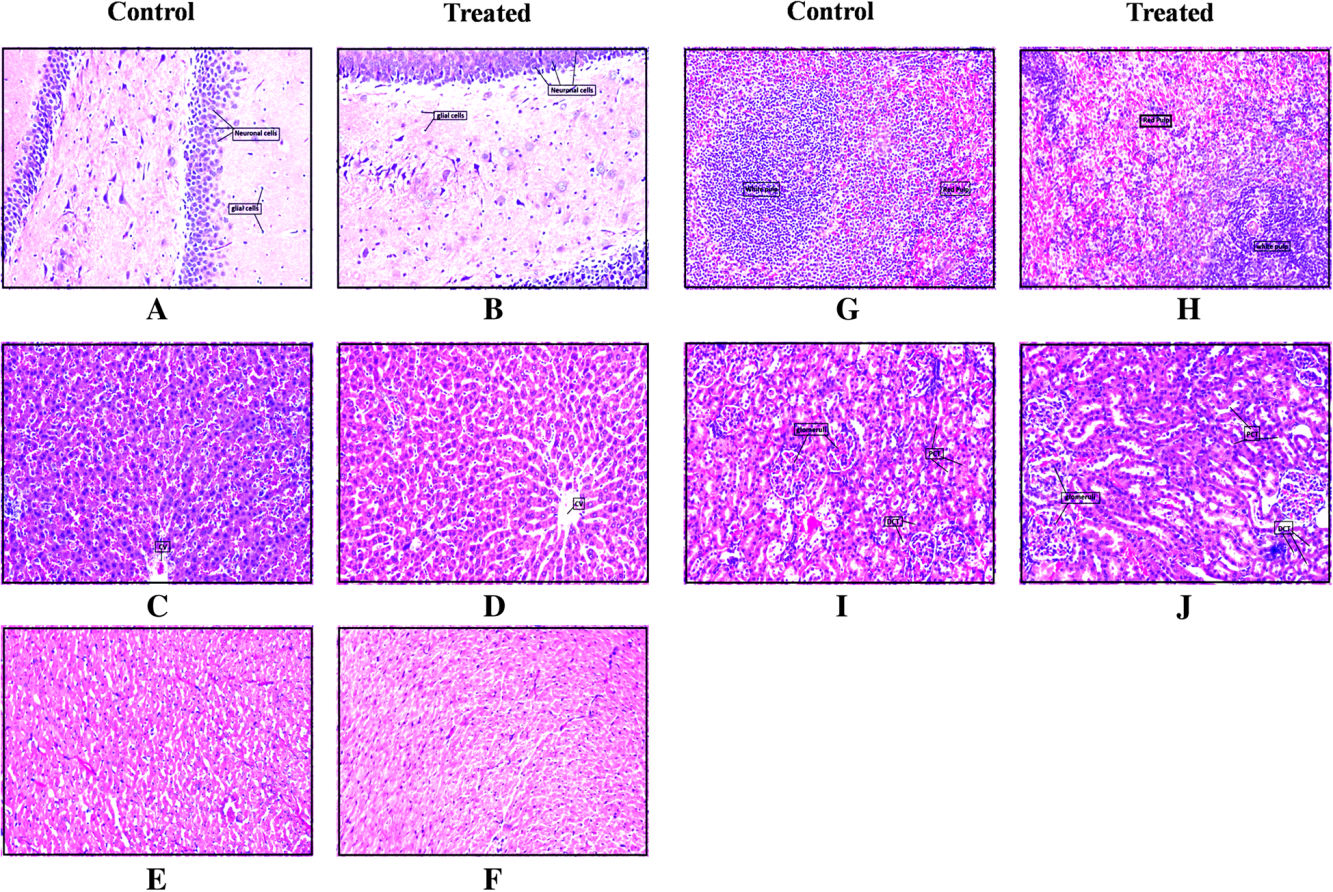
Effect of 1000 mg kg^−1^of Asdamarin on histology of vital organs of female rats. **a** and (**b**): brain; (**c**) and (**d**): liver; (**e**) and (**f**): heart; (**g**) and (**h**): spleen; (**i**) and (**j**): kidney. cv, central vein; DCT, distal convoluted tubule; PCT, proximal convoluted tubule

The histopathological examination of vital organs showed no toxic signs. The treatment with 1000 mg kg^−1^did not induce any changes in the cellular architecture of the examined tissues of male and female rats. Figures 5 and 6 shows the normal tissue morphology and absence of any gross lesions in organs.

## Discussion

We have investigated the efficacy of a combination of herbal extracts such as *F. asafoetida* and *S. marianum* (Asdamarin) in mitigating the delayed gastric emptying associated with functional dyspepsia, using phenol red method in rats. In accordance with Rome III criteria, FD is categorized based on the symptoms into postprandial distress syndrome (PDS) and epigastric pain syndrome (EPS) [4]. Gastric emptying is the natural process of clearing the content of the stomach after food consumption, and transferring to the small intestine [2]. PDS pathogenesis involves delay in gastric emptying and impaired gastric acclimatization [25]. Delayed gastric emptying and gastrointestinal motility are the main contributors to the gastrointestinal problems such as functional dyspepsia and nausea. Amelioration of gastrointestinal functions by mitigating the gastric emptying is an effective strategy to treat FD [25]. In the present study a 7-day pre-treatment with Asdamarin dose-dependently improved the gastric emptying rats. Gastric hypomotility involves dysfunction of serotonergic receptors 5- HT3/5- HT4 receptors [26]. It could be possible that the active constituents in Asdamarin modulate these receptors to exert its efficacy. We also found that Asdamarin significantly increased the intestinal transit in rats. Several prokinetic drugs of synthetic origin have similar mode of action [27]. However, the associated side effects of these medications cannot be ignored [28, 29]. In the light of these facts, the present study provide evidence on the efficacy of Asdamarin as a functional ingredient which could be explored in the treatment of FD.

We have further evaluated the toxicity of Asdamarin in rats. Single high dose (2000 mgkg^− 1^) administration of Asdamarin had no adverse effect on the rats after a 14-day observation. Sub-acute administration of Asdamarin did not induce any clinical signs of toxicity or mortality in the rats of either sex. There was no significant change in the food and water consumption by the rats throughout the study. Alterations in food and water consumption due to loss of appetite are often correlated to the decrement in body weight [30]. Furthermore, there was no significant change in the Asdamarin treated rats compared to the control group. Body weight changes are generally corroborated with the health status of an individual [31]. The data obtained from the present study clearly indicate that repeated oral consumption of Asdamarin does not have any adverse effect on the body metabolism. No significant changes were recorded in the relative organ weights of rats suggesting that Asdamarin had no effect on the normal growth. It was correlated well with the gross observation and the histopathology findings. There were no major haematological and biochemical changes in rats of either sex administered with the test doses of Asdamarin.

## Conclusion

Asdamarin is a unique herbal ingredient associating two well-known plants, together contributing to improvement in gastric emptying. The present study also provides preliminary evidence on the possible use of Asdamarin in treating the symptoms of FD such as delayed gastric emptying. No lethality or toxic signs were evident following acute and sub-acute administration of Asdamarin indicating the safety of the formulation.

## Data Availability

The data sets used and/or analysed during the current study available from the corresponding author on reasonable request.

## Abbreviations

ALT: Alanine aminotransferase
ANOVA: Analysis of variance
AST: Aspartate aminotransferase
CPCSEA: Committee for the purpose of control and supervision of experiments on animals
EDTA: Ethylenediaminetetraacetic acid
EPS: Epigastric pain syndrome
FD: Functional dyspepsia
GIT: Gastrointestinal transit
H&E: Hematoxylin and eosin
HPLC: High performance liquid chromatography
MCV: Mean corpuscular volume
OECD: Organization for economic cooperation and development
PDS: Postprandial distress syndrome
SSRI: Serotonin re-uptake inhibitors

## References

[1] Kawachi M, Matsunaga Y, Tanaka T, Hori Y, Ito K, Nagahama K (2011). Acotiamide hydrochloride (Z-338) enhances gastric motility and emptying by inhibiting acetylcholinesterase activity in rats. Eur J Pharmacol.

[2] Brook RA, Kleinman NL, Choung RS, Melkonian AK, Smeeding JE, Talley NJ (2010). Functional dyspepsia impacts absenteeism and direct and indirect costs. Clin Gastroenterol Hepatol.

[3] Aro P, Talley NJ, Ronkainen J, Storskrubb T, Vieth M, Johansson SE (2009). Anxiety is associated with uninvestigated and functional dyspepsia (Rome III criteria) in a Swedish population-based study. Gastroenterol.

[4] Choung RS, Locke GR, Schleck CD, Zinsmeister AR, Talley NJ (2007). Do distinct dyspepsia subgroups exist in the community? A population-based study. Am J Gastroenterol.

[5] Khonche A, Fallah Huseini H, Abdi H, Mohtashami R, Nabati F, Kianbakht S (2017). Efficacy of *Mentha pulegium* extract in the treatment of functional dyspepsia: a randomized double-blind placebo-controlled clinical trial. J Ethnopharmacol.

[6] Raveendra KR, Jayachandra SV, Sushma KR, Allan JJ, Goudar KS, et al. An extract of Glycyrrhiza glabra (GutGard) alleviates symptoms of functional dyspepsia: a randomized, double-blind, placebo-controlled study. Evid Based Complement Alternat Med. 2012:9. 10.1155/2012/216970 PMC3123991 21747893

[7] Karimi A, Majlesi M, Rafieian-Kopaei M (2015). Herbal versus synthetic drugs; beliefs and facts. J Nephropharmacol.

[8] Monkemuller K, Malfertheiner P (2006). Drug treatment of functional dyspepsia. World J Gastroenterol.

[9] Sahebkar A, Iranshahi M (2010). Biological activities of essential oils from the genus *Ferula* (Apiaceae). Asian Biomed.

[10] Duan H, Takaishi Y, Tori M, Takaoka S, Honda G, Ito M (2002). Polysulfide derivatives from Ferulafoetida. J Nat Prod.

[11] Mahendra P, Bisht S (2012). *Ferula asafoetida*: traditional uses and pharmacological activity. Pharmacogn Rev.

[12] Takeoka G, Takeoka GR, Guntert M, Engel K-H (2001). Volatile constituents of Asafoetida. Aroma active compounds in foods.

[13] Platel K, Srinivasan K (2000). Influence of dietary spices and their active principles on pancreatic digestive enzymes in albino rats. Nahrung.

[14] Gopi S, Amalraj A, Jude S, Varma K, Sreeraj TR, Haponiuk JT (2017). Preparation, characterization and anti-colitis activity of curcumin-asafoetida complex encapsulated in turmeric nanofiber. Mater Sci Eng C Mater Biol Appl.

[15] Vijayasteltar L, Jismy IJ, Joseph A, Maliakel B, Kuttan R, Krishnakumar IM (2017). Beyond the flavor: a green formulation of *Ferula asafetida* oleo-gum-resin with fenugreek dietary fibre and its gut health potential. Toxicol Rep.

[16] Bagheri SM, Hejazian SH, Dashti-R MH (2014). The relaxant effect of seed’s essential oil and oleo-gum-resin of *Ferula Assa-foetida* on isolated rat’s ileum. Ann Med Health Sci Res.

[17] Fatehi M, Farifteh F, Fatehi-Hassanabad Z (2004). Antispasmodic and hypotensive effects of *Ferula asafoetida* gum extract. J Ethnopharmacol.

[18] Natural Medicines Comprehensive (2012). Database: professional version. Milk thistle monograph.

[19] Khazim K, Gorin Y, Cavaglieri RC, Abboud HE, Fanti P (2013). The antioxidant silybin prevents high glucose-induced oxidative stress and podocyte injury in vitro and in vivo. Am J Physiol Renal Physiol.

[20] Aghazadeh S, Amini R, Yazdanparast R, Ghaffari SH (2011). Anti-apoptotic and anti-inflammatory effects of *Silybum marianum* in treatment of experimental steatohepatitis. Exp Toxicol Pathol.

[21] Yao J, Zhi M, Minhu C (2011). Effect of silybin on high-fat-induced fatty liver in rats. Braz J Med Biol Res.

[22] Camilleri M, Bueno L, Andresen V. Pharmacological, pharmacokinetic, and pharmacogenomic aspects of functional gastrointestinal disorders. Gastroenterol 2016; pii: S0016-5085(16)00220-1. 10.1053/j.gastro.2016.02.029 27144621

[23] Organization for Economic Cooperation and Development (OECD) guidelines for acute toxicity of chemicals. No. 425 (Adopted: 3 October 2008).

[24] Chandra P, Sachan N, Ghosh AK, Kishore K (2010). Acute and sub-chronic oral toxicity studies of a mineralo-herbal drug Amlena on experimental rats. Int J Pharm Res Innov.

[25] Mala KN, Thomas J, Syam DS, Maliakel B, Krishnakumar IM. Safety and efficacy of *Ferula asafetida* in functional dyspepsia: a randomized, double-blind, placebo-controlled study. Evid Based Compl Alt Med. 2018:11. 10.1155/2018/4813601 PMC6129344 30224930

[26] Poudel BK, Yu JY, Kwon YS (2015). The pharmacological effects of Benachio-F(®) on rat gastrointestinal functions. Biomol Ther (Seoul).

[27] Nikkhah Bodagh M, Maleki I, Hekmatdoost A (2018). Ginger in gastrointestinal disorders: a systematic review of clinical trials. Food Sci Nutr.

[28] Holtmann G, Talley NJ, Liebregts T, Adam B, Parow CN (2006). A placebo-controlled trial of itopride in functional dyspepsia. Engl J Med.

[29] Wysowski DK, Corken A, Gallo-Torres H, Talarico L, Rodriguez EM (2001). Postmarketing reports of QT prolongation and ventricular arrhythmia in association with cisapride and Food and Drug Administration regulatory actions. Am J Gastroenterol.

[30] Klaassen CD (2001). Casarett and Doull’s toxicology: the basic science of poisons.

[31] El Hilaly J, Israili ZH, Lyoussi B (2004). Acute and chronic toxicological studies of *Ajugaiva* in experimental animals. J Ethnopharmacol.

